# Supplementary material for: Causal interplay between lactose intolerance and gut microbiota: a combined bidirectional Mendelian randomization and in vivo validation study
Source: Front Nutr. 2026 Jun 1;13:1803337. doi: 10.3389/fnut.2026.1803337 (PMC13265576; doi:10.3389/fnut.2026.1803337)
Supplement: Supplementary file 1 [file Data_Sheet_1.zip › supplementary materials/Forward/leave one out cross validation/ebi-a-GCST90027814.finngen_R12_E4_LACTONAS.pdf]

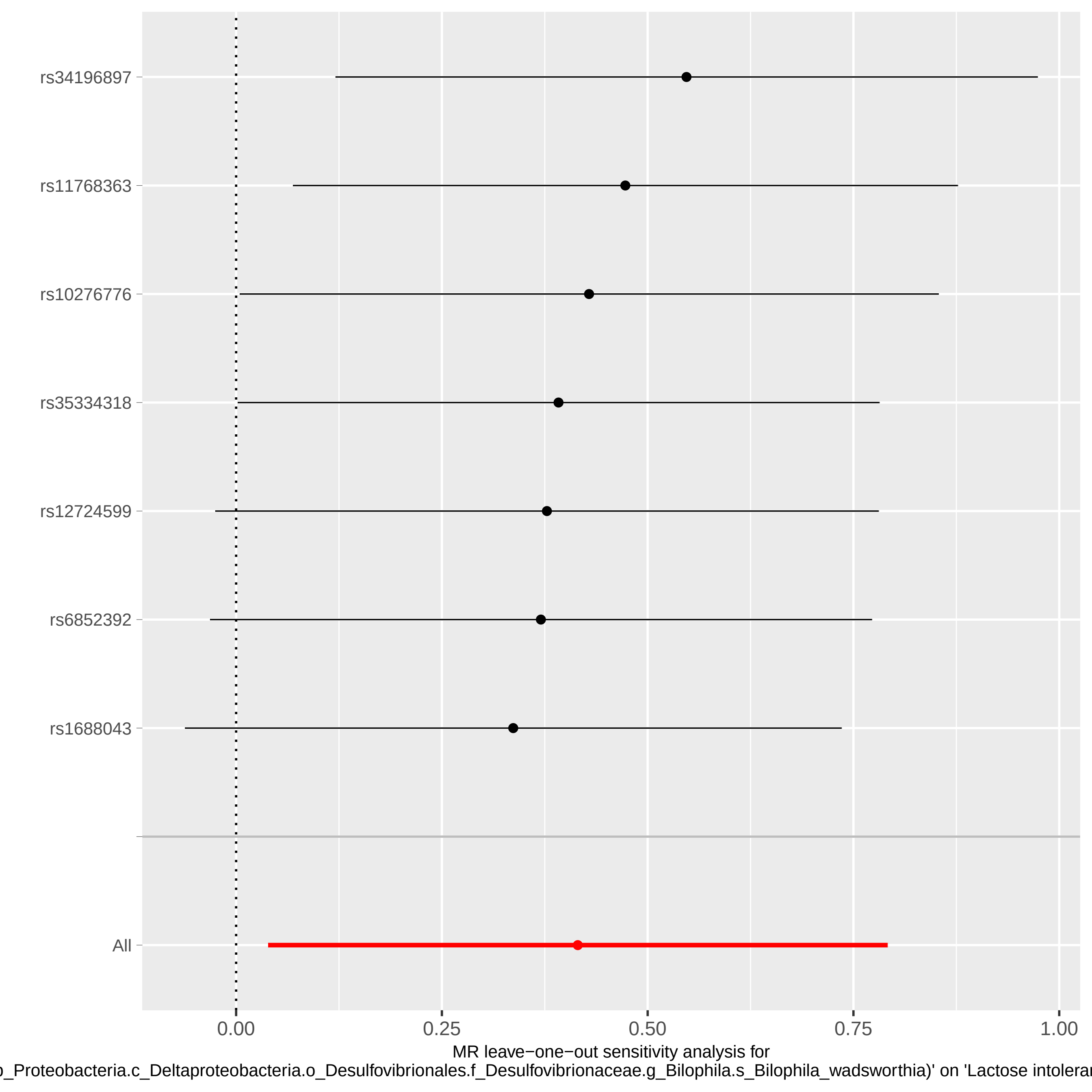
rs34196897
rs11768363
rs10276776
rs35334318
rs12724599
rs6852392
rs1688043
All
0.00
0.25
0.50
0.75
1.00
MR leave−one−out sensitivity analysis for
p_Proteobacteria.c_Deltaproteobacteria.o_Desulfovibrionales.f_Desulfovibrionaceae.g_Bilophila.s_Bilophila_wadsworthia)' on 'Lactose intoleran
